# miR-145-Mediated Tumor Suppression in Bladder Urothelial Carcinoma via Targeting the ADAMTS5/Wnt Signaling Axis

**DOI:** 10.7759/cureus.89796

**Published:** 2025-08-11

**Authors:** Jiang Wei, Zhang L Feng, Cheny Y Mao, Huang Xi, Fu Ming, Chen Jun

**Affiliations:** 1 Urology, Ezhou Central Hospital, Ezhou, CHN

**Keywords:** adamts5, bladder urothelial carcinoma, metastasis, mir-145, proliferation

## Abstract

Background: Bladder urothelial carcinoma (BLCA) is the most common urological tumor with high mortality. The present study aimed to investigate the role of miR-145 in the tumorigenesis of BLCA.

Materials and methods: The levels of miR-145 and ADAMTS5 were examined in BLCA specimens. BLCA cell lines T24 and 5637 were adopted for in vitro experiments. ADAMTS5 was verified as a downstream target of miR-145 using a luciferase reporter assay. Cell viability and apoptosis were detected using the CCK-8 assay and flow cytometry. Cell migratory and invasive capacities were examined by the transwell assay. The protein levels of ADAMTS5, E-cadherin, N-cadherin, and vimentin were analyzed by western blotting.

Results: miR-145 was found to be negatively correlated to the expression of ADAMTS5 in BLCA specimens. ADAMTS5 was identified as a direct target of miR-145. In addition, PCR and western blotting showed that miR-145 overexpression reduced ADAMTS5 levels, suppressed cell viability, and induced cell apoptosis, whereas miR-145 knockdown triggered the opposite result. miR-145 has also been verified to impede epithelial-mesenchymal transition (EMT) activation in tumor progression.

Conclusion: The present study revealed that miR-145 negatively regulates the expression of ADAMTS5. This regulatory effect of miR-145 may be associated with the Wnt/β‑catenin signaling pathway. Therefore, miR-145 has the potential to be used as a biomarker for predicting the progression of BLCA.

## Introduction

Bladder urothelial carcinoma (BLCA) is the most common primary malignancy in the urological system, accounting for approximately 5% of all urothelial malignancies [1,2]. It is well known for its high recurrence rate, rapid growth, high invasion capacity, and mortality. Despite improvements in therapeutic treatments, including surgical resection, chemotherapy, and immunotherapy, the median survival length for patients with BLCA remains 12-14 months [3,4]. Due to the lack of effective early-stage diagnostic methods, BLCA are difficult to distinguish from normal tissues and are radically resected. Furthermore, BLCA cells have been proven to be insensitive to radiotherapy and chemotherapy [5]. Therefore, seeking new effective targets underlying the progression of BLCA will shed new light on the development of diagnosis and targeted therapy.

MicroRNAs (miRNAs) include small non-coding RNAs composed of 21-25 nucleotides, with high conservation between species [6,7]. Newly discovered potential biomarkers, including miRNAs, are extremely promising because they have the ability to modulate target genes post-transcriptionally, thereby regulating the function of corresponding biological processes. Due to the unique biological characteristics, miRNAs can be used as diagnostic biomarkers [8]. In malignant tumors, miRNA can be used to assess the trend of tumor invasion, metastasis, or drug resistance as a prognostic indicator [9]. Therefore, it is essential to investigate the molecular mechanisms by which miRNAs are involved in the tumorigenesis and progression of BLCA.

In the present study, we observed that miR-145 markedly declined in human BLCA cells and samples. The molecular interactive signaling of miR-145 in BLCA has been tested, and its anti-tumor effect may be attributed to the negative regulation of ADAMTS5. In sum, the current study will shed new light to better understand the development and progress of BLCA.

## Materials and methods

Clinical specimens

This study is an in vitro molecular study using clinical samples and cell lines. The present study was performed with the approval of the Ethics Committee of Ezhou Central Hospital (approval number: ECH20240211). All patients provided written informed consent. The entire study was conducted in accordance with the Declaration of Helsinki and relevant national and international ethics guidelines. A total of 42 BLCA tissues and paired normal bladder tissues were surgically resected and immediately stored in −196°C liquid nitrogen until needed for subsequent experiments.

Cell lines and reagents

The human BLCA cell lines (T24 and 5637) and human benign urothelial line (SV-HUC-1) were purchased from NTCC Preservation Center (Beijing, China). All cells were cultured in RPMI-1640 medium (GIBCO, CA) at 37°C with 5% CO₂.

Transfection and plasmid construction

The miR-145 mimics, miR-145 inhibitor, ADAMTS5 siRNA, and negative controls were synthesized by the GeneChem Company (Shanghai, China). Lipofectamine 2000 reagent (Invitrogen, Waltham, MA, USA) was used for transfection, following the manufacturer's provided protocol.

The putative binding sites in the 3′ UTR of ADAMTS5 were cloned into the pGL3 vector to construct the luciferase plasmid. The ADAMTS5 3′ UTR sequence and pGL3 vector were purchased from GeneChem Company (Shanghai, China). The prediction of the miR-145 binding site was performed using the TargetScan database.

Luciferase reporter assays

For the luciferase reporter assay, cells were seeded in 24-well plates and co-transfected with luciferase vectors and either miR-145 mimics or NC for 48 hours. The activity of luciferase was measured using a dual luciferase reporter system (Promega, Madison, WI, USA).

Western blot

The total proteins were isolated from lysed cells (50 μg) and separated by 10% SDS-PAGE, then transferred to a polyvinylidene fluoride (PVDF) membrane. The bands were cultured with the primary antibodies against ADAMTS5 (ab45048, Abcam, UK), E-cadherin (#14472; Cell Signaling Technology, Danvers, MA, USA), vimentin (#5471; Cell Signaling Technology), and GAPDH (sc-47724, Santa Cruz Biotechnology, Inc., Santa Cruz, CA, USA) at 4 °C overnight, followed by horseradish peroxidase (HRP)-conjugated secondary antibodies. The protein level was quantified by Image-Pro Plus software.

Quantitative real-time PCR

The total RNA was isolated using Trizol reagent (Life Technologies, Waltham, MA, USA) and transcribed into complementary DNA using the RevertAid First Strand cDNA synthesis kit (Thermo Fisher Scientific, Inc., Waltham, MA, USA). Subsequently, cDNA synthesis was performed using reverse transcription PCR (Invitrogen, USA) with the SYBR Green method and GoScript™ Reverse Transcription Mix (Promega, random primers), followed by quantitative PCR on the AriaMx Real-Time PCR machine (Agilent Technologies, Santa Clara, CA, USA). GAPDH and U6 were chosen as the internal standard. The relative quantification was performed using the 2-∆∆Ct method. At least three technical repeats have been done for each pooled sample.

Cell proliferation assay

Cells were inoculated into 96-well microtiter plates and cultured after transfection. Subsequently, 10 μL of CCK-8 reagent was added at three time points (24, 48, and 96 hours). Cell density was measured using a microplate reader (BioTek Instruments, Winooski, VT, USA) at a wavelength of 450 nm.

Transwell assay

Cells were inoculated in a medium without FBS (fetal bovine serum) in the upper chamber coated with Matrigel. Media containing 10% FBS was added to the bottom chamber. After 48 hours of incubation, the cells were washed twice with PBS (phosphate buffered saline), then fixed with 4% paraformaldehyde and stained with hematoxylin and eosin. Cell counts were performed under a microscope across five random visual fields.

Statistical analysis

All data were analyzed using GraphPad Prism 5.0 (GraphPad Software, San Diego, CA, USA). Results were expressed by means ± standard deviation (SD). Each experiment was performed at least three times. Statistical analysis was performed using ANOVA with IBM SPSS Statistics for Windows, Version 20 (Released 2011; IBM Corp., Armonk, New York). A Student's t-test was used for comparison between the two groups. P < 0.05 was considered statistically significant.

## Results

ADAMTS5 is up-regulated in BLCA tissues

**Figure 1:**
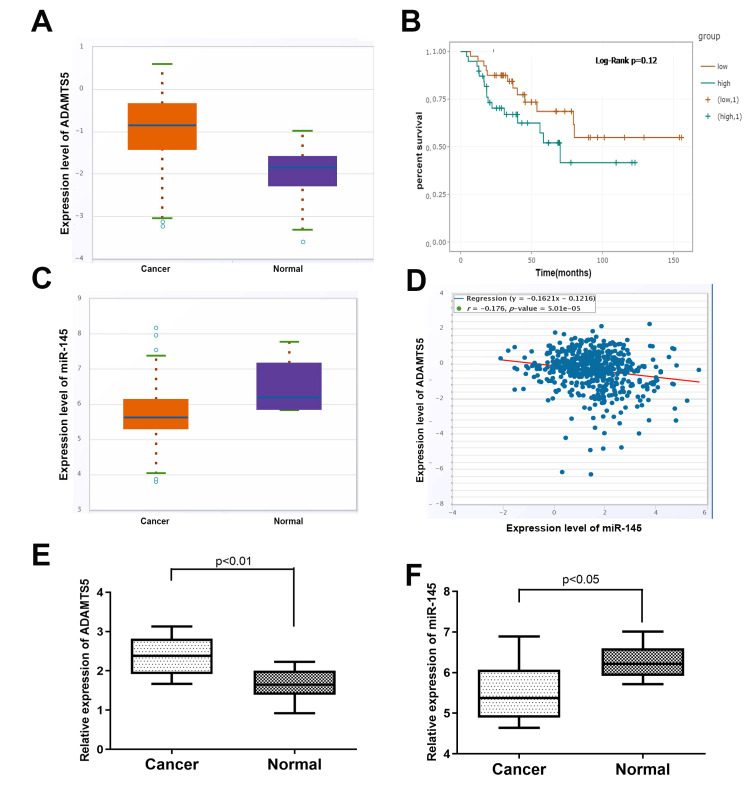
Verification of the miR-145/ADAMTS5 regulation axis. (A) The relative expression level of miR-145 in BLCA cell lines (T24 and 5637) and the human benign urothelial line (SV-HUC-1) (*P<0.05 vs. SV-HUC-1 group). (B) The manipulation of miR-145 expression by transfection with miR-145 inhibitors in T24 cells and transfection with miR-145 mimics in 5637 cells (*P<0.05 vs. NC group). (C) The predicted binding area of miR-145 and the ADAMTS5 3’UTR. (D, E) A dual luciferase reporter assay was performed in T24 and 5637 cells. Relative luciferase activities are presented. (F, G) Western blot and qRT-PCR analysis of ADAMTS5 expression in cells transfected with miR-145 inhibitors or mimics (*P<0.05 vs. NC group). NC: normal cells

The expression of ADAMTS5 was explored in Starbase ver3.0. As illustrated in Figure 1A, ADAMTS5 levels were significantly higher in BLCA tissues compared to normal tissues. Furthermore, survival analysis revealed that patients with low ADAMTS5 expression had better overall survival times than those with high ADAMTS5 levels (Figure 1B). Additionally, database analysis indicated that miR-145 expression in BLCA tissues was lower than in corresponding normal tissues (Figure 1C). qRT-PCR confirmed a similar trend in the tissues (Figures 1E, 1F). Through Pearson correlation analysis, we found that the expression of miR-145 was negatively correlated with ADAMTS5 (Figure 1D).

ADAMTS5 is a direct target of miR-145

**Figure 2:**
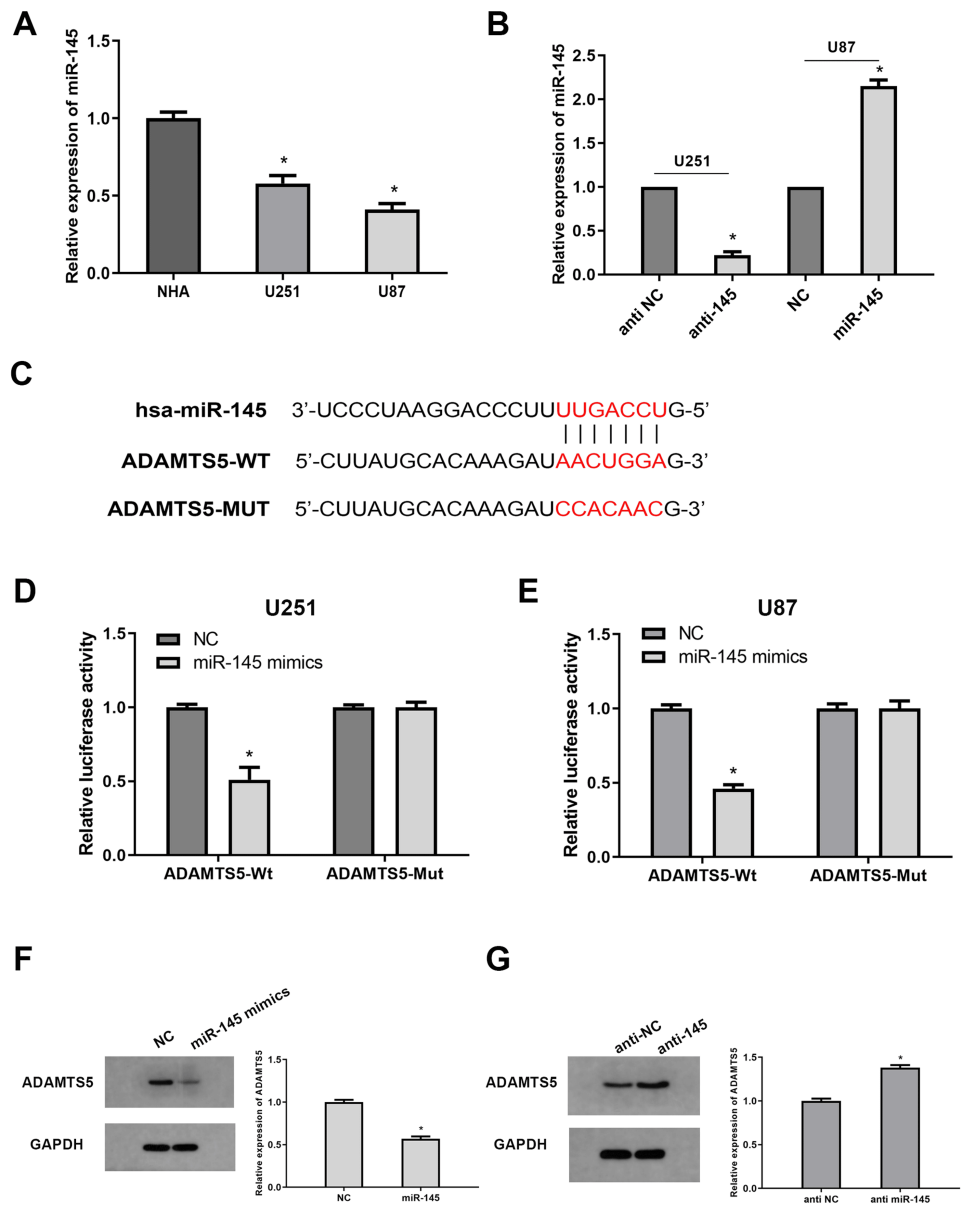
Verification of the miR-145/ADAMTS5 regulation axis. (A) The relative expression level of miR-145 in BLCA cell lines (T24 and 5637) and human benign urothelial line (SV-HUC-1) (*P<0.05 vs. SV-HUC-1 group). (B) The manipulaion of miR-145 expression by transfection with miR-145 inhibitors in T24 cells and transfection with miR-145 mimics in 5637 cells (*P<0.05 vs. NC group). (C) Predicted binding area of miR-145 and the ADAMTS5 3’UTR. (D, E) Dual Luciferase reporter assay was performed in T24 and 5637 cells. Relative luciferase activities are presented. (F, G) Western blot and qRT-PCR analysis of ADAMTS5 expression in cells transfected with miR-145 inhibitors or mimics (*P<0.05 vs. NC group). NC: normal cells

The correlation between miR-145 and ADAMTS5 was subsequently studied. As shown by qRT-PCR, compared with normal cells, miR-145 was significantly downregulated in BLCA cells (Figure 2A). Then, T24 and 5637 cells were transfected with miR-145 inhibitor or miR-145 mimic to obtain miR-145 silenced or activated cells (Figure 2B). Western blot data showed that the expression of ADAMTS5 in miR-145 silenced cells was moderately increased, and the expression in miR-145 overexpressing cells was significantly decreased (Figures 2C, 2D). Additionally, the potential binding site of miR-145 on ADAMTS5 was examined using a dual luciferase assay (Figure 2E). The results showed that the reporter activity of cells transfected with miR-145 mimic and wild-type ADAMTS5 3'UTR decreased significantly, while this suppressive effect was abolished in the ADAMTS MUT group (Figures 2F, 2G). As mentioned above, all these results indicate that miR-145 can negatively regulate the expression of ADAMTS5 by directly binding to it.

miR-145 regulates the biological behavior of BLCA in vitro

**Figure 3:**
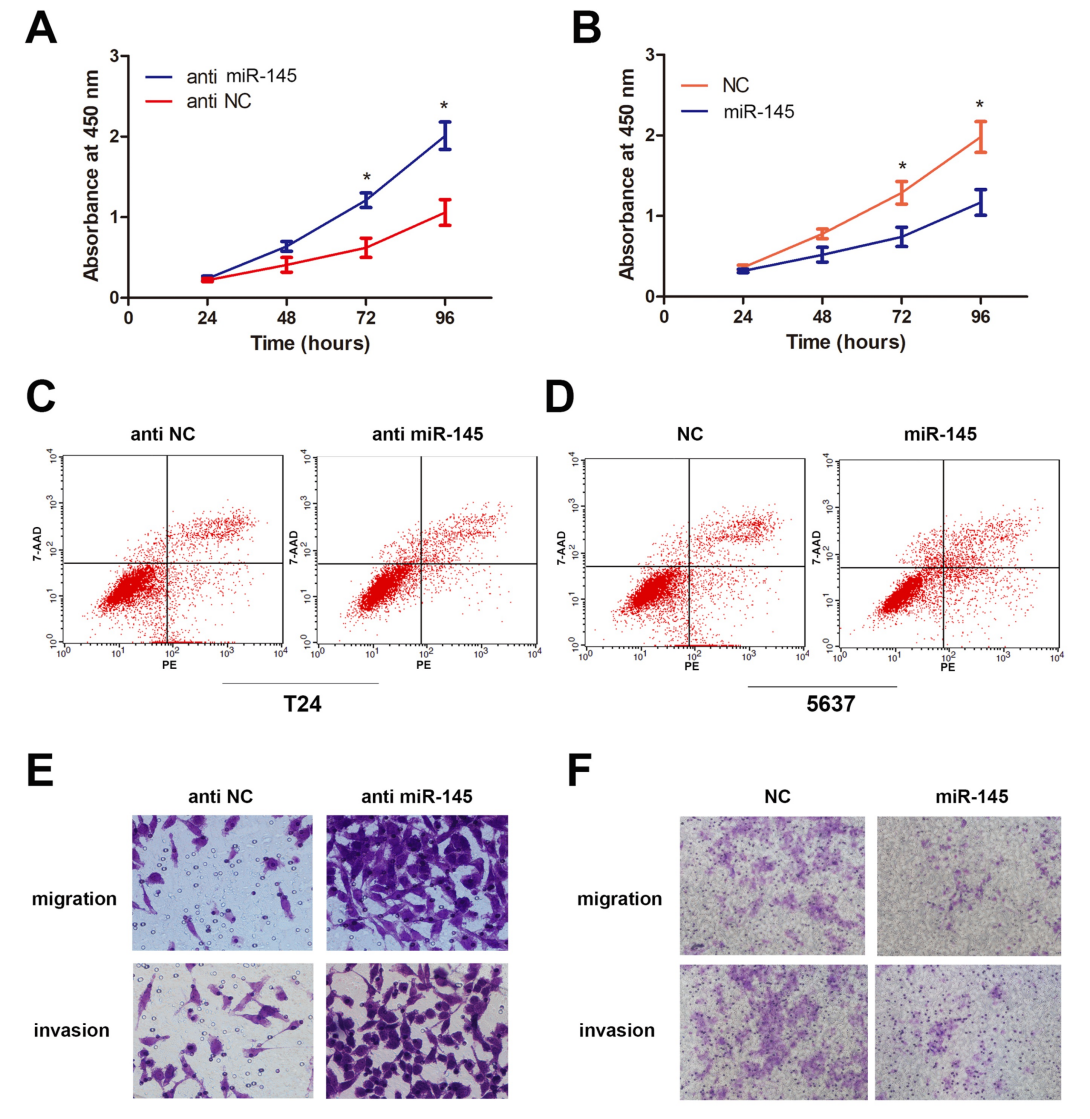
The regulatory role of miR-145 in BLCA in vitro. (A, B) CCK-8 assays were performed to determine cell viability. Data were collected at 24, 48, 72, and 96 hours after transfection (*P<0.05 vs. NC group). (C, D) Flow cytometry was performed to measure cell apoptosis (*P<0.05 vs. NC group). (E, F) Transwell migration and invasion assays were performed to assess the migratory and invasive abilities. The cell number was counted in five random fields at 200× magnification (*P < 0.05 vs. NC group). NC: normal cells

To determine the role of miR-145 in the proliferation of BLCA, a CCK-8 assay was conducted. Data showed that miR-145 silencing significantly promoted the proliferation of BLCA cells in a time-dependent manner, while overexpression of miR-145 resulted in a reduction of cell viability, indicating that miR-145 can effectively inhibit the proliferation of BLCA cells (Figures 3A, 3B). A wound healing assay was performed to detect cell migration. As the results showed, compared with the NC (normal cells) group, miR-145 activation led to a significant decrease in cell migration, while miR-145 silence abrogated this inhibitory effect (Figures 3C, 3D). The transwell assay showed that the migration and invasion ability of the miR-145 overexpression group significantly declined, while it was significantly enhanced in the miR-145 silence group (Figures 3E, 3F). In summary, the above findings confirm that miR-145 functions as an oncogene in BLCA cells.

miR-145 activates the ADAMTS5/Wnt/β‑catenin signaling pathway

**Figure 4:**
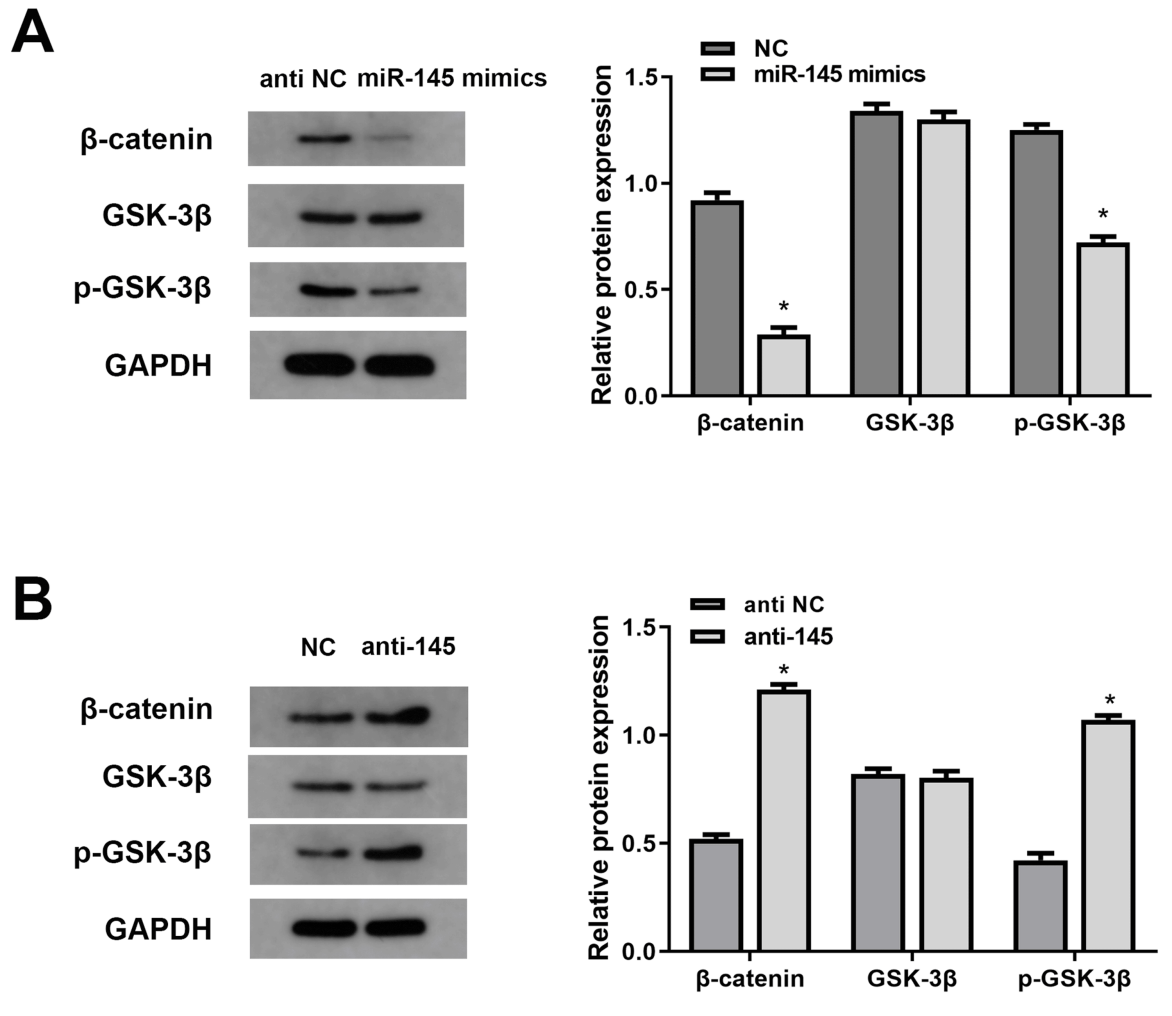
Regulation of miR-145 on the Wnt/β-catenin signaling pathway. (A, B) The protein levels of β-catenin, p-GSK-3β, and GSK-3β in cells transfected with miR-145 inhibitors or miR-145 mimics. GAPDH was used as an internal control (*P<0.05 vs. NC group). NC: normal cells

It has been reported that ADAMTS5 regulates osteoarthritis injury through Wnt/β-catenin signaling [10]. To examine whether miR-145 regulates the Wnt/β-catenin signaling pathway via ADAMTS5, we analyzed the phosphorylation levels of GSK-3β and β-catenin. Western blotting showed that miR-145 overexpression dramatically reduced the level of β‑catenin and phosphorylated GSK-3β while hardly changed the protein expression of GSK-3β (Figures 4A, 4B). On the contrary, miR-145 silencing led to an activation of β‑catenin and phosphorylated GSK-3β. These data indicated that miR-145 could inactivate the Wnt/β‑catenin signaling pathway, which may be an important factor in the tumorigenesis of BLCA cells.

## Discussion

BLCA is a highly aggressive urological tumor characterized by invasive growth, high recurrence, and mortality. The accumulating literature has revealed that the abnormal expression of miRNAs can regulate gene expression and is involved in various biological processes of human malignant tumors [11-13]. The present study demonstrated that miR-145 plays an important role in the progression of BLCA. Multiple studies have reported that miR-145 is dysregulated in a variety of cancers. These studies all suggest that the abnormal expression of miR-145 is closely related to the malignant progression and poor prognosis of cancers [14-17]. In the current study, miR-145 expression was found to be significantly down-regulated in BLCA cells and clinical tissues. MiR-145 down-expression is highly correlated with distant metastasis, suggesting that miR-145 may play a key role in BLCA tumorigenesis. Here, by activating or silencing miR-145 in BLCA cells, we found that overexpression of miR-145 led to a significant decrease in cell growth and invasion in vitro, whereas silencing of miR-145 abrogated this effect in BLCA cells. These findings suggest that miR-145 acts as a tumor suppressor in BLCA.

To further investigate the regulatory mechanism of miR-145, the potential target of miR-145 in BLCA was predicted by means of bioinformatics prediction. As the prediction showed, ADAMTS5 may be a downstream target of miR-145. The expression of ADAMTS5 was markedly inhibited in miR-145 overexpressing cells. The double luciferase assay demonstrated that miR-145 overexpression led to a significant reduction in the luciferase reporter activity of cells expressing the wild-type ADAMTS5, whereas it had no effect on cells expressing the mutant ADAMTS5 vector. Therefore, our study verified that miR-145 directly targets ADAMTS5.

ADAMTS5 is a member of the ADAMTS (A Disintegrin And Metalloproteinase with Thrombospondin Motif) family, which shares the metalloproteinase domain with matrix metalloproteinases (MMPs) [18,19]. The ADAMTS family has been found to regulate multiple biological processes, including extracellular matrix maintenance, angiogenesis, and embryogenesis [20,21]. Among the total 19 members, ADAMTS5 is well known for its capacity in cartilage degradation [22]. Recent studies found that ADAMTS5 is involved in the tumorigenesis and progression of various cancers [23,24]. It has been reported that ADAMTS5 is overexpressed in BLCA cells and contributes to BLCA cell invasion through the cleavage of brevican [25]. However, little is known about the regulatory correlation between miRNA and ADAMTS5 in BLCA. Our findings indicate that ADAMTS5 is a functional target of miR-145. Its mRNA level was negatively correlated with the expression of miR-145 in clinical tissues.

## Conclusions

In conclusion, overexpression of miR-145 is closely related to the progression and metastasis of BLCA. Our study confirmed that miR-145 acts as a tumor suppressor in the occurrence and development of BLCA by down-regulating ADAMTS5. Collectively, our current study suggested that miR-145 has the potential to be a promising therapeutic target for BLCA.

## References

[1] Siegel RL, Giaquinto AN, Jemal A (2024). Cancer statistics, 2024. CA Cancer J Clin.

[2] Jubber I, Ong S, Bukavina L (2023). Epidemiology of bladder cancer in 2023: a systematic review of risk factors. Eur Urol.

[3] Guo J, Ma X, Liu D (2024). A distinct subset of urothelial cells with enhanced EMT features promotes chemotherapy resistance and cancer recurrence by increasing COL4A1-ITGB1 mediated angiogenesis. Drug Resist Updat.

[4] Yang Z, Chen Q, Dong S (2024). Hypermethylated TAGMe as a universal-cancer-only methylation marker and its application in diagnosis and recurrence monitoring of urothelial carcinoma. J Transl Med.

[5] Yu Z, Xiong Z, Ma J (2024). Prognostic and clinicopathological significance of systemic immune-inflammation index in upper tract urothelial carcinoma: a meta-analysis of 3911 patients. Front Oncol.

[6] Hirschberger S, Hinske LC, Kreth S (2018). MiRNAs: dynamic regulators of immune cell functions in inflammation and cancer. Cancer Lett.

[7] Li Q, Wang H, Peng H (2019). MicroRNAs: key players in bladder cancer. Mol Diagn Ther.

[8] Seyhan AA (2024). Trials and tribulations of microRNA therapeutics. Int J Mol Sci.

[9] Deng Q, Hu H, Yu X (2019). Tissue-specific microRNA expression alters cancer susceptibility conferred by a TP53 noncoding variant. Nat Commun.

[10] Hao X, Wu X (2024). SP1‑mediated ADAMTS5 transcription promotes IL‑1β‑induced chondrocyte injury via Wnt/β‑catenin pathway in osteoarthritis. Mol Med Rep.

[11] Xie Y, Liu X, Hu T, Wang W (2020). miR-302e suppresses glioma progression by targeting VEGFA. Cancer Manag Res.

[12] Zottel A, Šamec N, Kump A (2020). Analysis of miR-9-5p, miR-124-3p, miR-21-5p, miR-138-5p, and miR-1-3p in glioblastoma cell lines and extracellular vesicles. Int J Mol Sci.

[13] Aloizou AM, Pateraki G, Siokas V (2020). The role of MiRNA-21 in gliomas: hope for a novel therapeutic intervention?. Toxicol Rep.

[14] Su Y, Yu T, Wang Y, Huang X, Wei X (2021). Circular RNA circDNM3OS functions as a miR-145-5p sponge to accelerate cholangiocarcinoma growth and glutamine metabolism by upregulating MORC2. Onco Targets Ther.

[15] Liu X, Zhong L, Jiang W, Wen D (2021). Repression of circRNA_000684 inhibits malignant phenotypes of pancreatic ductal adenocarcinoma cells via miR-145-mediated KLF5. Pancreatology.

[16] Lu H, Zheng G, Gao X, Chen C, Zhou M, Zhang L (2021). Propofol suppresses cell viability, cell cycle progression and motility and induces cell apoptosis of ovarian cancer cells through suppressing MEK/ERK signaling via targeting circVPS13C/miR-145 axis. J Ovarian Res.

[17] Mu Y, Liu WJ, Bie LY, Mu XQ, Zhao YQ (2021). Blocking VRK2 suppresses pulmonary adenocarcinoma progression via ERK1/2/AKT signal pathway by targeting miR-145-5p. Eur Rev Med Pharmacol Sci.

[18] Mead TJ, Apte SS (2018). ADAMTS proteins in human disorders. Matrix Biol.

[19] Verma P, Dalal K (2011). ADAMTS-4 and ADAMTS-5: key enzymes in osteoarthritis. J Cell Biochem.

[20] Kelwick R, Desanlis I, Wheeler GN, Edwards DR (2015). The ADAMTS (a disintegrin and metalloproteinase with thrombospondin motifs) family. Genome Biol.

[21] Nandadasa S, Foulcer S, Apte SS (2014). The multiple, complex roles of versican and its proteolytic turnover by ADAMTS proteases during embryogenesis. Matrix Biol.

[22] Santiago Cal, Alvaro J Obaya, Llamazares M, Garabaya C, Quesada V, López-Otín C (2002). Cloning, expression analysis, and structural characterization of seven novel human ADAMTSs, a family of metalloproteinases with disintegrin and thrombospondin-1 domains. Gene.

[23] Zhu Z, Xu J, Wu X, Lin S, Li L, Ye W, Huang Z (2021). In silico identification of contradictory role of ADAMTS5 in hepatocellular carcinoma. Technol Cancer Res Treat.

[24] Malvia S, Bagadi SA, Pradhan D (2019). Study of gene expression profiles of breast cancers in Indian women. Sci Rep.

[25] Nakada M, Miyamori H, Kita D (2005). Human glioblastomas overexpress ADAMTS-5 that degrades brevican. Acta Neuropathol.

