# Correction: miR-145-Mediated Tumor Suppression in Bladder Urothelial Carcinoma via Targeting the ADAMTS5/Wnt Signaling Axis

**DOI:** 10.7759/cureus.c300

**Published:** 2025-09-19

**Authors:** Jiang Wei, Zhang L Feng, Cheny Y Mao, Huang Xi, Fu Ming, Chen Jun

**Affiliations:** 1 Urology, Ezhou Central Hospital, Ezhou, CHN

This article has been corrected as follows:

- Results section, first subheader: "ADAMTS5 is **downregulated** in BLCA tissues" corrected to ADAMTS5 is **up-regulated** in BLCA tissues"
- Results section, last paragraph: "Western blotting showed that miR-145 **knockdown** dramatically reduced..." corrected to "Western blotting showed that miR-145 **overexpression** dramatically reduced..."
- Results section, last paragraph: "On the contrary, miR-145 **overexpression** led to an activation..." corrected to "On the contrary, miR-145 **silencing** led to an activation..."
- Discussion section, first paragraph: "In the current study, miR-145 expression was found to be significantly **up-regulated** in BLCA cells and clinical tissues. MiR-145 **overexpression** is highly correlated with distant metastasis, suggesting that miR-145 may play a key role in BLCA tumorigenesis. Here, by activating or silencing miR-145 in BLCA cells, we found that overexpression of miR-145 led to a significant **increase** in cell growth and invasion in vitro, whereas silencing of miR-145 abrogated this effect in BLCA cells. These findings suggest that miR-145 acts as **an oncogene** in BLCA." corrected to "In the current study, miR-145 expression was found to be significantly **down-regulated** in BLCA cells and clinical tissues. MiR-145 **down-expression** is highly correlated with distant metastasis, suggesting that miR-145 may play a key role in BLCA tumorigenesis. Here, by activating or silencing miR-145 in BLCA cells, we found that overexpression of miR-145 led to a significant **decrease** in cell growth and invasion in vitro, whereas silencing of miR-145 abrogated this effect in BLCA cells. These findings suggest that miR-145 acts as **a tumor suppressor** in BLCA."
- Conclusions section: "Our study confirmed that miR-145 acts as **an oncogene** in the occurrence..." corrected to "Our study confirmed that miR-145 acts as **a tumor suppressor** in the occurrence..."
- Revised Figure 4 in accordance with above corrections
- Revise legend for Figure 2 in accordance with above corrections

